# Blood Flow Restriction Training in Knee Arthroplasty: A Systematic Review of Current Evidence on Postoperative Muscle Strength and Function

**DOI:** 10.3390/medicina61101879

**Published:** 2025-10-20

**Authors:** Bassem Tiss, Saoussen Layouni, Hela Ghali, Halil İbrahim Ceylan, Iheb Nticha, Sonia Jemni, Raul Ioan Muntean, Nicola Luigi Bragazzi, Ismail Dergaa

**Affiliations:** 1 Physical Medicine and Rehabilitation Department, Sahloul University Hospital, Faculty of Medicine of Sousse, University of Sousse, Sousse 4000, Tunisia; ettisbassem@gmail.com (B.T.); layouni.saoussen@famso.u-sousse.tn (S.L.); iheb.nticha@gmail.com (I.N.); sonia.jemni@famso.u-sousse.tn (S.J.); 2 Department of Preventive and Community Medicine, Sahloul University Hospital, Faculty of Medicine of Sousse, University of Sousse, Sousse 4000, Tunisia; hela.ghali@famso.u-sousse.tn; 3 Physical Education of Sports Teaching Department, Faculty of Sports Sciences, Atatürk University, Erzurum 25240, Türkiye; 4 Department of Physical Education and Sport, Faculty of Law and Social Sciences, University “1 Decembrie 1918” of Alba Iulia, 510009 Alba Iulia, Romania; 5 Laboratory for Industrial and Applied Mathematics (LIAM), Department of Mathematics and Statistics, York University, Toronto, ON M3J 1P3, Canada; robertobragazzi@gmail.com; 6 High Institute of Sport and Physical Education of Ksar Said, University of Manouba, Manouba 2010, Tunisia; phd.dergaa@gmail.com; 7 Physical Activity Research Unit, Sport and Health (UR18JS01), National Observatory of Sports, Tunis 1003, Tunisia; 8 Department of Social Sciences, High Institute of Sports and Physical Education of Kef, University of Jendouba, El Kef 8189, Tunisia

**Keywords:** blood flow restriction, knee arthroplasty, muscle strength, prehabilitation, postoperative recovery, rehabilitation, total knee replacement, vascular occlusion

## Abstract

*Background and Objectives:* Knee arthroplasty often leads to marked postoperative muscle weakness, with strength losses of up to 62% in the first month, contributing to functional impairment and patient dissatisfaction. Blood flow restriction (BFR) training, which combines low-load exercise with partial vascular occlusion, has shown promise in enhancing muscle strength across musculoskeletal conditions and may represent a valuable rehabilitation strategy for this vulnerable population. This review aimed to systematically evaluate the effectiveness and safety of BFR training in improving muscle strength and functional outcomes following knee arthroplasty. *Materials and Methods:* This systematic review was prospectively registered within PROSPERO (CRD420250652404) and conducted according to PRISMA guidelines. PubMed, Embase, and Cochrane Library were searched through February 2025 for randomized controlled trials (RCTs) investigating BFR training in knee arthroplasty patients. Study selection, data extraction, and risk of bias assessment (RoB 2 tool) were performed independently by two reviewers. Eligible trials reported muscle strength and/or functional outcomes as primary or secondary endpoints. *Results*: Four RCTs, including 148 patients undergoing total knee arthroplasty (mean age: 67 ± 6.5 years), met the inclusion criteria. All applied preoperative BFR training for 4–8 weeks with heterogeneous protocols. Two trials demonstrated significant improvements in muscle strength (1 RM leg press, 1 RM knee extension; large effect sizes) and functional outcomes (6 min walk test, 30 s sit-to-stand; earlier recovery), favoring BFR. The remaining studies showed no significant between-group differences, though moderate-to-large effect sizes generally favored BFR training. No adverse events were reported. *Conclusions*: Prehabilitation with BFR training shows considerable potential to enhance early postoperative muscle strength and functional recovery in patients undergoing knee arthroplasty, particularly when compared with usual care lacking structured preoperative intervention. The evidence to date suggests that BFR is a safe and well-tolerated strategy, offering an alternative for patients who cannot perform high-load resistance training. Its favorable safety profile, combined with the potential to accelerate functional recovery, highlights the promise of BFR for reducing rehabilitation costs and healthcare utilization. Nonetheless, larger, high-quality RCTs with standardized protocols and extended follow-up are required to confirm these preliminary findings and establish clear clinical guidelines for their implementation.

## 1. Introduction

Knee arthroplasty is among the most frequently performed orthopedic interventions worldwide. In 2018, France reported 113,600 primary knee arthroplasty procedures, corresponding to a 32.2% increase compared to 2012 [1,2]. At the same time, in the United States, primary total knee arthroplasty (TKA) volume in 2019 reached 480,958 procedures [3]. Similarly, Germany saw a 32.4% increase in annual TKA volume between 2005 and 2018 [4]. According to the OECD (2023), knee replacement surgery continues to expand across most European countries, with volumes projected to increase substantially in the coming decades [5].

This surgical procedure is primarily indicated for end-stage osteoarthritis and, increasingly, in trauma-associated contexts, delivering substantial pain relief and functional improvement for millions of patients annually [6]. However, despite ongoing advances in implant technology and refined surgical techniques, a non-negligible proportion of patients, with estimates ranging from 8% to 30%, report dissatisfaction following TKA, with functional limitations and residual symptoms representing a primary concern [7,8,9]. That said, more recent systematic reviews suggest that the true average rate of dissatisfaction may be lower: for example, DeFrance and Scuderi (2023) found a mean dissatisfaction rate of about 10%, and meta-analyses report rates around 10% [10]. The discrepancy in reported ranges likely reflects heterogeneity in study populations, definitions of dissatisfaction, timing of assessment, and inclusion of complications or revision cases. The economic burden extends beyond direct surgical costs, as prolonged rehabilitation periods and suboptimal functional recovery contribute to increased healthcare utilization and reduced productivity [11,12].

The physiopathology of postoperative muscle dysfunction following knee arthroplasty involves complex interactions between surgical trauma, inflammatory responses, and neuromuscular adaptation patterns [13]. Quadriceps muscle weakness emerges as a predominant concern, with strength losses reaching up to 62% in the first postoperative month, significantly exceeding those observed in other orthopedic procedures [13,14]. This profound weakness stems from multiple mechanisms, including arthrogenic muscle inhibition, where joint mechanoreceptor dysfunction disrupts normal motor unit recruitment patterns [14]. Additional contributing factors include surgical denervation, inflammatory mediator release, and prolonged immobilization, creating a cascade of events that perpetuate muscle atrophy and functional impairment [13,14]. Clinically, the manifestations extend beyond isolated strength deficits, encompassing altered movement patterns, reduced proprioception, and compromised balance control, collectively impacting activities of daily living and quality of life measures [14]. Furthermore, the relationship between muscle weakness and functional impairment appears bidirectional, with poor functional outcomes reinforcing muscle deconditioning and creating a cycle of progressive deterioration [13].

It is important to emphasize that prehabilitation aims to optimize functional reserve and muscle strength before surgery, thereby mitigating the magnitude of postoperative decline, whereas postoperative programs primarily focus on restoring function and mobility after surgical trauma.

The management of knee osteoarthritis (KOA), the primary indication for TKA, extends beyond surgical strategies. Conservative approaches represent the first-line treatment and include patient education, weight management, activity modification, therapeutic exercise, and physical therapy modalities [15], often supplemented by pharmacological interventions such as oral non-steroidal anti-inflammatory drugs (NSAIDs) or intra-articular corticosteroid and hyaluronic acid injections [16,17]. These interventions aim to alleviate pain, improve function, and delay the need for surgical management. However, when conservative measures fail to provide sufficient relief, and functional disability progresses, surgical intervention in the form of total or partial knee arthroplasty becomes the definitive treatment, offering substantial pain reduction and improvements in quality of life for patients with end-stage KOA [6,18].

Despite an extensive body of scholarly literature, several critical research gaps still limit our understanding of optimal rehabilitation strategies following knee arthroplasty. First, there exists insufficient evidence regarding the effectiveness of novel rehabilitation approaches that can be safely implemented in elderly, frail populations who may not tolerate traditional high-load resistance training protocols [19]. Second, limited research has investigated preoperative interventions that could potentially mitigate postoperative muscle weakness and functional decline, with most studies focusing on postoperative rehabilitation strategies [20]. Third, current rehabilitation protocols often fail to address the unique physiological constraints of the immediate postoperative period, when pain, inflammation, and movement restrictions limit traditional strengthening approaches [21]. Fourth, there is an inadequate investigation of interventions that can provide comparable benefits to high-load resistance training while minimizing mechanical stress on healing tissues [22]. Fifth, existing studies have not sufficiently explored the safety profile and tolerability of innovative rehabilitation techniques in the specific population of knee arthroplasty patients, who often present with multiple comorbidities and advanced age [21,23]. Sixth, there remains a limited understanding of interventions that can be effectively implemented across diverse healthcare settings with varying resource availability and expertise levels [24].

Given these research gaps, this systematic review aimed to evaluate the effectiveness and safety of blood flow restriction training (BFR) for improving muscle strength and functional outcomes following knee arthroplasty. The primary objective was to synthesize current evidence from randomized controlled trials (RCTs) regarding the impact of BFR training on postoperative muscle strength recovery. Secondary objectives included assessing functional outcome improvements, safety profiles, and optimal implementation protocols for this intervention.

## 2. Methods

### 2.1. Protocol Registration and Reporting Guidelines

This systematic review protocol was prospectively registered within the International Prospective Register of Systematic Reviews (PROSPERO; registration number CRD420250652404, registered on 14 February 2025, prior to database searching and data extraction) and is accessible at the following link: https://www.crd.york.ac.uk/PROSPERO/view/CRD420250652404 (accessed on 24 February 2025). The review was conducted and reported in accordance with the PRISMA 2020 guidelines [25]. A preliminary literature search was conducted on 21 February 2025, to confirm the absence of existing systematic reviews addressing the same research question.

### 2.2. Eligibility Criteria

Eligibility criteria were defined using the PICOS framework [26]. Population (P) included patients who underwent knee arthroplasty (total or partial). Intervention (I) of interest was BFR training implemented at any time point relative to surgery. Comparisons (C) included control groups receiving usual care, alternative interventions, or sham interventions. Outcomes (O) included muscle strength measurements and/or functional performance assessments as primary or secondary endpoints. Concerning study design (S), only RCTs were included to ensure the highest level of evidence. Exclusion criteria encompassed studies including patients with other types of knee surgery, review articles, case reports, case series, expert opinions, and non-English publications. No restrictions were imposed regarding study setting, country of origin, or publication date.

### 2.3. Search Strategy

A comprehensive search strategy was developed and executed across three major databases: PubMed, Embase, and the Cochrane Library. The search was conducted on 28 February 2025, using a combination of Medical Subject Heading (MeSH) terms in PubMed, EMTREE terms in Embase, and controlled vocabulary in the Cochrane Library, supplemented by free-text keywords to maximize sensitivity. Boolean operators (AND/OR) were systematically employed to combine relevant search terms. The search strategy incorporated terms related to BFR training (including “KAATSU training”, “occlusion training”, “ischemic training”, “vascular occlusion training”, and “tourniquet training”), knee arthroplasty procedures (including “total knee arthroplasty”, “knee replacement”, “partial knee arthroplasty”, and “unicompartmental knee arthroplasty”), and relevant outcomes (including “muscle strength”, “functional outcomes”, “recovery of function”, and “physical performance”). No filters were applied during the initial search to ensure comprehensive coverage. Reference lists of included studies were manually screened to identify additional relevant publications (Table 1).

### 2.4. Study Selection Process

All retrieved records were imported into Rayyan, a web-based systematic review management tool, where duplicate entries were systematically identified and removed [27]. The remaining studies underwent a two-stage selection process. First, titles and abstracts were independently screened by two reviewers against the predefined eligibility criteria. Second, full-text articles of potentially relevant studies were independently assessed by the same two reviewers for final inclusion. Discrepancies at any stage were resolved through discussion, and when consensus could not be reached, a third reviewer was consulted. To quantify the level of agreement between reviewers during the screening and selection phases, inter-rater reliability was calculated using Cohen’s kappa (κ), which demonstrated substantial agreement (κ = 0.90), indicating high consistency between independent assessments. A standardized screening form was used throughout to document decisions and reasons for exclusion.

### 2.5. Data Extraction Protocol

Data extraction was independently conducted by two reviewers using a standardized, pilot-tested extraction form specifically designed for this review. Extracted information included study characteristics (design, setting, duration), participant demographics (sample size, age, sex distribution, underlying pathology), intervention details (BFR protocol, control group procedures, treatment duration), outcome measures (muscle strength assessment methods, functional performance tests), and results (primary and secondary outcomes, adverse events). Each reviewer extracted data independently, and discrepancies were resolved through discussion or, when necessary, by consulting a third reviewer.

### 2.6. Risk of Bias Assessment

Risk of bias was assessed using the revised Cochrane Risk of Bias tool (RoB 2) for RCTs [28]. This tool evaluates bias at the level of specific outcomes and across five domains: (1) bias arising from the randomization process, (2) bias due to deviations from intended interventions, (3) bias due to missing outcome data, (4) bias in measurement of the outcome, and (5) bias in selection of the reported result. Each domain was judged as ‘low risk,’ ‘some concerns,’ or ‘high risk,’ and a domain-specific summary was generated for each included study and outcome. The Physiotherapy Evidence Database (PEDro) scale [29] was additionally applied to provide descriptive information on methodological quality, but the main interpretation of study quality was based on the RoB 2 assessments.

### 2.7. Data Synthesis and Analysis

Due to heterogeneity in study designs, intervention protocols, and outcome measures, a narrative synthesis approach was employed rather than quantitative meta-analysis. Results were organized by outcome categories (muscle strength, functional performance, safety) and presented using standardized mean differences and confidence intervals, where available. Effect sizes were interpreted using Cohen’s conventions (small: 0.2, medium: 0.5, large: 0.8). Heterogeneity between studies was assessed qualitatively based on differences in population characteristics, intervention protocols, and outcome measurement methods.

## 3. Results

### 3.1. Study Selection and Characteristics

**Figure 1:**
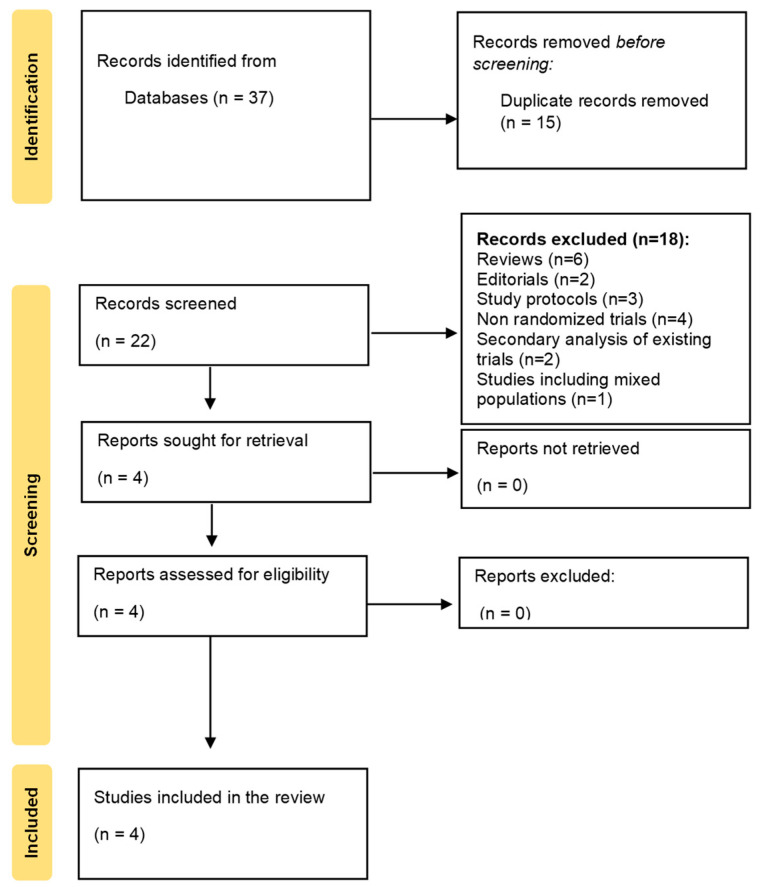
PRISMA 2020 flow diagram of the literature search and selection process. PRISMA, Preferred Reporting Items for Systematic Reviews and Meta-Analyses.

A total of 37 records were identified through database searching. After removing 15 duplicates, 22 records were screened based on title and abstract. Of these, 18 full-text articles were excluded for failing to meet the eligibility criteria, leaving four studies that met all inclusion criteria. Therefore, four RCTs met the inclusion criteria and were included in the final analysis [30,31,32,33]. The PRISMA 2020 flow diagram (Figure 1) illustrates the selection process. A detailed list of excluded studies, along with the reasons for exclusion, is provided in Supplementary Table S1.

The included studies, published between 2021 and 2024, collectively enrolled 148 patients undergoing TKA (Table 2). The study populations demonstrated similar baseline characteristics, with a mean age of 67 ± 6.53 years and sex distribution of 62 males and 86 females. All participants had end-stage KOA as the primary indication for surgery. Notably, all four studies implemented BFR training as a prehabilitation intervention during the preoperative period, with intervention durations ranging from 4 to 8 weeks before surgery.

### 3.2. Risk of Bias Assessment

**Figure 2:**
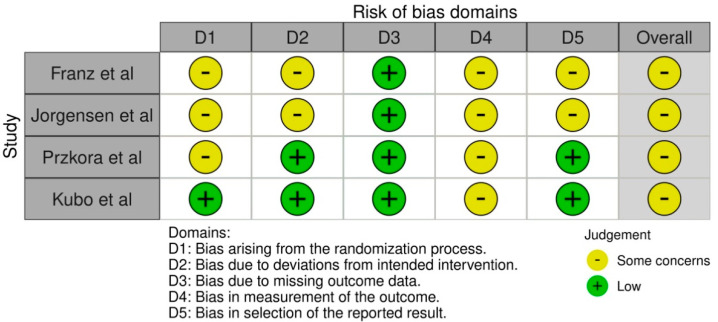
Risk of bias assessment using the revised Cochrane Risk of Bias tool (RoB 2) [30,31,32,33].

The RoB 2 assessments are summarized in Figure 2. Across the five domains, most studies demonstrated ‘some concerns’ in the domains of randomization and deviations from intended interventions, whereas outcome measurement and missing data were generally judged at ‘low risk’. Selective reporting was unclear in two studies that lacked pre-registered protocols [31,33]. A structured matrix of domain-specific judgments is provided for each study. PEDro scores ranged from 4 to 6/10, reflecting moderate methodological quality, but these ratings are provided only as descriptive indicators, while our main interpretation is based on the RoB 2 domain assessments.

### 3.3. Intervention Characteristics and Protocols

Considerable heterogeneity existed in BFR training protocols across studies (Table 2). Pressure determination methods varied significantly: two studies used limb occlusion pressure (LOP) calculations [30,31], one employed an automated inflator system [32], and one applied arbitrary pressure settings (100–120 mmHg) [33]. Cuff application protocols differed: three studies used thigh-only cuffs [30,31,32], whereas Kubo et al. applied cuffs to both the thigh and the calf [33]. Exercise protocols showed variation: three studies combined BFR with resistance training at 30% of maximum resistance or voluntary contraction [30,31,32], while one study incorporated cycling ergometer training [30]. Control group designs varied: two studies used no-intervention control groups [31,32], while two employed sham interventions with low-pressure cuffs [30,33].

### 3.4. Muscle Strength Outcomes

#### 3.4.1. Significant Improvements in Muscle Strength

Franz et al. reported statistically significant improvements in quadriceps strength favoring the BFR group across all measured parameters [30]. Participants demonstrated higher strength values than both the control and active control groups preoperatively and maintained superior leg strength up to 6 months post-surgery. The magnitude of these improvements was substantial, with large effect sizes observed consistently across timepoints (1 RM leg press, SMD = 0.82, 95% CI 0.41–1.23). Similarly, Jørgensen et al. showed that the BFR group significantly outperformed controls in 1 RM knee extension and leg press at nearly all timepoints, and reported clinically meaningful gains in functional performance, with improvements exceeding the minimal clinically significant difference (MCID) for both the 30 STS (+3.1 repetitions, 95% CI 1.9–4.2) and the 6 MWT (+58 m, 95% CI 30–85) [31]. However, not all outcomes were consistently significant: isometric knee flexor strength in Jørgensen et al. failed to reach statistical significance despite moderate-to-large effect sizes, while Przkora et al. and Kubo et al. did not identify significant between-group differences in isometric strength or functional outcomes [32,33].

#### 3.4.2. Non-Significant Strength Outcomes

In contrast, Kubo et al. and Przkora et al. did not observe statistically significant between-group differences in muscle strength measures [32,33]. However, Kubo et al. noted similar patterns of quadriceps strength progression in both the BFR and control groups, which performed low-intensity resistance training with slow movement and tonic force generation [33]. Another study by Przkora et al. found comparable decreases in peak torque between groups, suggesting that BFR training may have provided some protective effect against the expected post-surgical strength decline [32].

### 3.5. Functional Performance Outcomes

#### 3.5.1. Significant Functional Improvements

Franz et al. observed significant improvements in functional performance exclusively in the BFR group [30]. The 6 min walk test demonstrated substantial improvements, with large effect sizes at most time points, while the 30 s sit-to-stand test showed faster improvements than control groups. These functional benefits appeared to manifest earlier in the BFR group (at 3 weeks prehabilitation) and persisted longer through the early postoperative period.

#### 3.5.2. Mixed Functional Outcomes

Jørgensen et al. reported mixed results regarding functional outcomes, with no statistically significant between-group differences despite moderate-to-large effect sizes favoring BFR training both preoperatively and at 3 months postoperatively [31]. Interestingly, by 12 months postoperatively, the control group showed superior performance on functional measures, suggesting that the initial benefits of BFR training may not be sustained long-term without continued intervention.

Kubo et al. and Przkora et al. found no significant differences in functional test performance between groups [32,33]. However, Przkora et al. observed that the BFR group showed less decline in Short Physical Performance Battery scores compared to controls, though both groups demonstrated similar declines in 6 min walk test performance [32].

### 3.6. Safety Profile and Adverse Events

A particularly notable finding across the included studies was that no adverse events directly attributable to BFR training were reported [30,31,32,33]. However, it should be noted that one trial listed adverse events as ‘not reported,’ and therefore, the absence of reporting cannot be equated with the lack of occurrence. Among the studies that provided safety data, no complications, such as thromboembolism, excessive muscle damage, or cardiovascular events, were documented. Furthermore, where reported, haemodynamic parameters (e.g., blood pressure and heart rate responses) remained within safe and expected ranges during training, supporting the feasibility of implementing BFR protocols in this preoperative population characterized by advanced age, multiple comorbidities, and joint pathology.

## 4. Discussion

The principal findings of this systematic review provide preliminary evidence supporting the potential benefits of BFR training as a prehabilitation strategy for patients undergoing knee arthroplasty. Among the four included RCTs, two demonstrated statistically significant improvements in muscle strength and functional outcomes favoring BFR training [30,31]. At the same time, the remaining studies showed non-significant results with moderate-to-large effect sizes generally favoring the intervention [32,33]. Importantly, no adverse events were reported across all studies, establishing a favorable safety profile for this intervention in the knee arthroplasty population. The findings suggest that BFR training may be particularly beneficial when implemented as prehabilitation, rather than usual care without structured preoperative intervention, offering a promising approach to enhance early postoperative recovery.

### 4.1. Individual Result Interpretation

#### 4.1.1. Muscle Strength Improvements

The significant improvements in muscle strength observed in two studies [30,31] align with established physiological mechanisms underlying BFR training’s effectiveness. The combination of low-load exercise with partial vascular occlusion creates a hypoxic environment that stimulates multiple pathways promoting muscle hypertrophy and strength gains [34]. These mechanisms include increased production of hypoxia-inducible factors, enhanced muscle angiogenesis, elevated growth factor expression (particularly VEGF and IGF-1), and accumulation of metabolites that stimulate muscle protein synthesis [22]. The observed improvements in 1 RM knee extension and leg press, with large effect sizes [31], suggest that BFR training can effectively target the quadriceps, which is most susceptible to weakness following knee arthroplasty [13].

The clinical significance of these strength improvements extends beyond statistical measures, as even modest increases in muscle strength can translate to meaningful functional benefits in older adults [19]. Previous research in anterior cruciate ligament reconstruction populations has demonstrated similar benefits, with BFR training producing strength gains comparable to those of high-load resistance training while placing lower mechanical stress on healing tissues [19]. This finding is particularly relevant for patients undergoing knee arthroplasty, who often present with significant joint pathology and may not tolerate traditional high-intensity strengthening protocols [35].

#### 4.1.2. Functional Performance Outcomes

The functional improvements observed in the 6 min walk test and 30 s sit-to-stand test [30] represent clinically meaningful outcomes that directly impact patient independence and quality of life. The 6 min walk test serves as a valid indicator of cardiovascular fitness and functional capacity, with improvements reflecting enhanced endurance and mobility [36]. Similarly, the sit-to-stand test assesses lower extremity strength and power, which are essential for activities of daily living [37]. The faster improvements observed in the BFR group suggest that prehabilitation may accelerate functional recovery trajectories, potentially reducing rehabilitation time and healthcare costs [11].

However, the mixed functional outcomes observed across studies [31,32,33] highlight the complex relationship between muscle strength and functional performance in this population. The observation that control groups eventually achieved superior functional performance at 12 months post-surgery [31] suggests that initial prehabilitation benefits may require ongoing intervention to maintain long-term advantages. This finding underscores the importance of comprehensive rehabilitation programs that extend beyond the immediate preoperative period [38].

#### 4.1.3. Safety Profile and Tolerability

The consistent absence of adverse events across all studies [30,31,32,33] represents a crucial finding that distinguishes BFR training from traditional high-load resistance training approaches. This safety profile is significant given the vulnerable characteristics of the knee arthroplasty population, including advanced age, multiple comorbidities, and pre-existing cardiovascular concerns [20]. The lack of reported complications such as thromboembolism, rhabdomyolysis, or cardiovascular events supports the potential for widespread clinical implementation [7].

Previous systematic reviews in other populations have similarly demonstrated the safety of BFR training when appropriate protocols are followed [24]. The pressure parameters used in the included studies (40–80% of arterial occlusion pressure) align with established safety guidelines that minimize risks while maximizing therapeutic benefits [21]. This safety profile becomes particularly relevant when considering that many knee arthroplasty patients may be contraindicated for high-intensity exercise due to cardiovascular limitations or joint pathology [24].

### 4.2. Non-Significant Results 

The non-significant results observed in two studies [32,33] provide important insights into factors that may influence the effectiveness of BFR training. Post hoc power calculations suggest that these studies may have been underpowered to detect clinically meaningful differences, particularly given the modest sample sizes (*n* = 10 and *n* = 22, respectively). The clinical importance of the observed effect sizes, despite statistical non-significance, suggests that larger, adequately powered studies might reveal statistically significant benefits.

Alternative explanations for non-significant results include differences in control-group interventions; Kubo et al. compared BFR training with an active control group performing low-intensity resistance training [33]. This comparison may have reduced between-group differences compared with studies using usual-care control groups [30,31]. Additionally, variations in pressure application protocols, exercise modalities, and intervention duration may have influenced outcomes, highlighting the need for standardized implementation guidelines [39].

### 4.3. Practical Implications and Applications

#### 4.3.1. Clinical Practice Implications

Healthcare providers should consider BFR training as a viable prehabilitation option for knee arthroplasty patients, particularly those unable to tolerate high-load resistance training due to pain, frailty, or comorbidities [22]. The intervention’s safety profile and potential for early functional recovery make it especially suitable for elderly patients with multiple health conditions. Implementation should follow established pressure calculation protocols (40–80% of arterial occlusion pressure) and include appropriate monitoring for adverse events [21].

Physical therapists and exercise physiologists can integrate BFR training into existing prehabilitation programs, potentially reducing treatment time while maintaining therapeutic benefits [19]. The technique’s relatively simple implementation requirements make it accessible across diverse healthcare settings, from hospital-based programs to outpatient rehabilitation centers [40].

#### 4.3.2. Health System Implications

The potential for enhanced early postoperative recovery through BFR training prehabilitation may reduce healthcare utilization and costs. Faster functional recovery could translate into shorter rehabilitation duration, fewer follow-up visits, and a reduced risk of complications related to prolonged immobility [41]. However, since no formal economic analyses were performed in the included studies, these potential cost benefits should be considered hypothetical and warrant confirmation in future cost-effectiveness research. Health systems may cautiously consider incorporating BFR training into prehabilitation protocols, particularly for high-volume knee arthroplasty programs [11]. Still, implementation should be accompanied by a structured evaluation of both clinical and economic outcomes. Policy implications include the need for training programs to ensure safe and effective delivery across healthcare providers, with quality improvement initiatives focusing on standardized protocols and outcome monitoring to maximize benefits while minimizing risks [20].

#### 4.3.3. Patient Care Implications

For patients, BFR training offers a potentially more tolerable alternative to traditional high-intensity strengthening exercises while maintaining therapeutic benefits. The intervention’s safety profile and reduced mechanical stress may improve adherence to prehabilitation programs, particularly among elderly patients with joint pain or functional limitations [42]. Patients should be educated about the technique’s benefits and safety measures to encourage participation in preoperative conditioning programs [43].

Shared decision-making should incorporate discussion of BFR training as a prehabilitation option, particularly for patients with contraindications to high-load exercise or preferences for lower-intensity interventions [37].

### 4.4. Limitations

This systematic review has several limitations that should be acknowledged when interpreting the findings. First, the small number of included studies (*n* = 4) and the limited total sample size (*n* = 148) constrain the strength of evidence and the generalizability of findings. The modest sample sizes of individual studies may have led to underpowered analyses, particularly for detecting minor but clinically meaningful effects. Second, considerable heterogeneity existed in BFR training protocols, including pressure determination methods (e.g., LOP 40–60% vs. fixed pressures 100–120 mmHg), cuff application sites (thigh vs. thigh + calf), exercise modalities, and intervention durations. This heterogeneity precludes definitive conclusions about optimal implementation parameters and may have contributed to inconsistent results across studies. Third, the comparators used across trials were heterogeneous (‘usual care,’ sham-BFR, or active low-load strength training). Fourth, all included studies used BFR training as prehabilitation, limiting conclusions about the timing and effectiveness of its postoperative application. The exclusive focus on preoperative intervention may not reflect the full potential of BFR training throughout the perioperative continuum. Fifth, although no adverse events were reported, one study did not explicitly report adverse events (‘NR’), and the small sample sizes mean that rare but serious events such as thromboembolism cannot be definitively excluded. Thus, safety findings should be interpreted with caution. Sixth, the overall risk of bias assessment revealed methodological concerns across all studies, including inadequate randomization procedures, lack of blinding, and limited protocol registration. In addition, no formal assessment of reporting bias or statistical heterogeneity was performed, which may further limit the robustness of our findings. Seventh, the relatively short follow-up periods in most studies (ranging from 2 weeks to 6 months) prevent assessment of long-term benefits and sustainability of treatment effects. Finally, potential language and publication biases must be acknowledged, as the review excluded non-English articles and did not include trial registry searches.

For each limitation, specific mitigation strategies were employed where possible, including comprehensive database searches, standardized data extraction procedures, and transparent reporting of study characteristics and results. Future research should address these limitations through larger, adequately powered studies with standardized protocols, extended follow-up periods, and formal assessment of reporting biases.

## 5. Conclusions

We found preliminary evidence that BFR training can be a beneficial prehabilitation strategy for patients undergoing knee arthroplasty. Two of four studies included in this review showed significant improvements in muscle strength and functional outcomes, with moderate-to-large effect sizes generally favoring BFR training. The lack of adverse events suggests a favorable safety profile for this intervention in the knee arthroplasty population. Healthcare providers should consider BFR training as a safe and well-tolerated option for prehabilitation in knee arthroplasty patients, especially those who are unable to perform high-load resistance training due to age, frailty, or comorbidities. To implement this training effectively, healthcare providers should follow established pressure calculation protocols and monitor patients closely. The potential for improved early postoperative recovery could reduce healthcare utilization and costs, making it a valuable addition to standard prehabilitation programs for high-volume knee arthroplasty services. BFR training offers a potentially more tolerable alternative to traditional high-intensity exercise while maintaining therapeutic benefits, potentially improving adherence to preoperative conditioning programs. To establish optimal implementation guidelines and assess long-term effectiveness, researchers need to conduct high-quality RCTs with larger sample sizes, standardized protocols, and extended follow-up periods. Comparative studies evaluating BFR training against established high-load resistance programs within structured prehabilitation frameworks will help clarify its clinical utility. Investigating the implementation of postoperative BFR training, the optimal timing for intervention initiation, and the cost-effectiveness of the intervention are also key research priorities. 

In conclusion, this systematic review adds to the growing evidence base supporting innovative rehabilitation approaches for patients undergoing knee arthroplasty, providing insights into safe and effective alternatives to traditional high-load exercise protocols. The findings support the development of evidence-based prehabilitation programs that can be implemented across diverse healthcare settings to improve postoperative outcomes.

## Figures and Tables

**Table 1:** Research strategy across databases.

Pubmed	Query	Results
#4	#1 AND #2 AND #3	12
#1	(“Blood Flow Restriction Training”[MeSH] OR “Blood Flow Restriction Exercise” OR “BFR Therapy” OR “BFR Therapies” OR “KAATSU Training” OR “Occlusion Training” OR “Ischemic Training” OR “Ischemic Exercise” OR “Vascular Occlusion Training” OR “Tourniquet Training”)	1491
#2	(“Arthroplasty, Replacement, Knee”[MeSH] OR “Arthroplasty, Knee Replacement” OR “Knee Replacement Arthroplasty” OR “Knee Arthroplasty” OR “Knee Replacement” OR “Total Knee Arthroplasty” OR “Total Knee Replacement” OR “TKA” OR “TKR” OR “Partial Knee Arthroplasty” OR “Unicompartmental Knee Arthroplasty” OR “Unicondylar Knee Arthroplasty” OR “Unicompartmental Knee Replacement” OR “Partial Knee Replacement”)	51,916
#3	(“Muscle Strength” [MeSH] OR “Quadriceps Strength” OR “Lower Limb Strength” OR “Muscle Power” OR “Postoperative Muscle Weakness” OR “Muscle Endurance” OR “Recovery of Function” [MeSH] OR “Functional Outcome” OR “Functional Performance” OR “Activities of Daily Living” [MeSH] OR “ADL” OR “Gait” [MeSH] OR “Mobility Limitation” [MeSH] OR “Mobility” OR “Physical Function” OR “Functional Independence” OR “Walking Ability” OR “Sit-to-Stand Performance” OR “Timed Up and Go” OR “TUG” OR “Balance”)	877,859
Embase	Query	
#4	#1 AND #2 AND #3	13
#1	(‘blood flow restriction training’/exp OR ‘blood flow restriction exercise’ OR ‘BFR therapy’ OR ‘BFR therapies’ OR ‘KAATSU training’ OR ‘occlusion training’ OR ‘ischemic training’ OR ‘ischemic exercise’ OR ‘vascular occlusion training’ OR ‘tourniquet training’)	1046
#2	(‘knee arthroplasty’/exp OR ‘arthroplasty, knee replacement’ OR ‘knee replacement arthroplasty’ OR ‘knee arthroplasty’ OR ‘knee replacement’ OR ‘total knee arthroplasty’ OR ‘total knee replacement’ OR ‘TKA’ OR ‘TKR’ OR ‘partial knee arthroplasty’ OR ‘unicompartmental knee arthroplasty’ OR ‘unicondylar knee arthroplasty’ OR ‘unicompartmental knee replacement’ OR ‘partial knee replacement’)	70,865
#3	(‘muscle strength’/exp OR ‘quadriceps strength’ OR ‘lower limb strength’ OR ‘muscle power’ OR ‘postoperative muscle weakness’ OR ‘muscle endurance’ OR ‘functional outcome’/exp OR ‘recovery of function’/exp OR ‘activities of daily living’/exp OR ‘mobility’/exp OR ‘gait’/exp OR ‘functional performance’ OR ‘physical function’ OR ‘functional independence’ OR ‘walking ability’ OR ‘sit-to-stand performance’ OR ‘timed up and go’ OR ‘TUG’ OR ‘balance’)	854,915
Cochrane Library	Query	
	(“Blood Flow Restriction Training” OR “Blood Flow Restriction Exercise” OR “BFR Therapy” OR “BFR Therapies” OR “KAATSU Training” OR “Occlusion Training” OR “Ischemic Training” OR “Ischemic Exercise” OR “Vascular Occlusion Training” OR “Tourniquet Training”) AND (“Total Knee Arthroplasty” OR “Total Knee Replacement” OR “TKA” OR “TKR” OR “Knee Replacement” OR “Knee Arthroplasty” OR “Partial Knee Replacement” OR “Unicompartmental Knee Arthroplasty” OR “Unicondylar Knee Arthroplasty”) AND (“Muscle Strength” OR “Quadriceps Strength” OR “Lower Limb Strength” OR “Muscle Power” OR “Postoperative Muscle Weakness” OR “Muscle Endurance” OR “Functional Outcome” OR “Recovery of Function” OR “Functional Performance” OR “Activities of Daily Living” OR “ADL” OR “Gait” OR “Mobility” OR “Physical Function” OR “Functional Independence” OR “Walking Ability” OR “Sit-to-Stand Performance” OR “Timed Up and Go” OR “TUG” OR “Balance”) IN Title Abstract Keyword	12

**Table 2:** Summary results of all included studies.

Source	Study Design	Setting	Sample Characteristics	Description of the Intervention	BFR Protocol	Function and/or Muscle Strength Measurement Tools	Results
[30]	Single-blinded RCT with parallel group	**Location of the study:** Germany **Enrollment period: NR**	***n* = 30** total, ***n* = 10** control group, ***n* = 10** active control (AC) group, ***n* = 10** BFR group **mean age:** 63.5 ± 8.1 years **sex distribution:** 18 males and 12 females **Inclusion criteria:** patients with end-stage knee OA undergoing unilateral TKA	6 weeks before a TKA The control group had a standard clinical treatment (surgery and 3 weeks inpatient post op rehabilitation) The active control group had a prehabilitation program with sham-BFR (tourniquet in alternation between both legs with a standard pressure of 20 mmHg while training with a cycling ergometer) The BFR group had the same rehabilitation protocol as the active control group, but loaded with 40% of the individual LOP **Assessment timepoints:** Baseline 3w prehab Preop 3m-post op 6m-post op	2 sessions of 50 min a week Pressure was 40% of the individual LOP, alternating between both legs during cycling ergometer training For each session, BFR was applied 3 times, for 1 min in the first week and 6 min in the sixth week, in each leg -Cuffs were applied to the thigh	**Muscle strength:** 6 RM test of leg extension and leg curl **Function:** Active knee joint mobility (ROM) 30 STS 6 MWT	**Muscle strength:** At **Pre-op** assessment, the BFR group showed significantly higher values in all strength measures than the other groups Significant differences in leg strength between the BFR group and other groups during the **overall** **postoperative period** concerning the operated and the non-operated leg **Function:** Only the BFR group showed a significant improvement of **6 MWT** with a large effect size in all timepoints except for 3m post op Both AC and BFR groups showed improvements of the **30 STS**, but BFR improvements were faster (occurring at 3w prehab), without a drop at 3m postop, unlike the AC group. No significant changes in the **ROM** **Adverse event: None**
[31]	Multicenter, randomized, assessor-blinded, controlled trial	**Location of the study:** Denmark **Enrollment period:** from September 2019 to October 2022	***n* = 86** (total), ***n* = 42** (control group) and ***n* = 44** (BFR group) **mean age:** 66.6 ± 6.62 years **sex distribution:** 37 males and 49 females **Inclusion criteria:** patients aged ≥ 50 years scheduled for primary TKA for advanced knee OA	**8 weeks before a primary TKA** **The control group: 2 to 3 weeks of** usual preoperative care, including information and physical activity, and usual postop care **The BFR group:** same as the control group, but including pre-op BFR training **Assessment time points:** baseline (12 weeks preop) Pre-op 3m-post op 12m-post op	3 sessions a week 10 min warm-up on an ergometer bike **THEN** **4** sessions of **Leg press** exercise and **four** sessions of **Leg extension** exercise The first session: **30** reps The second and third: **15** reps The fourth until volitional **contraction failure** 5 min rest between the two exercises **Pressure** was 60% of the LOP The **load** was 30% 1 RM, but increased if the fourth session surpassed 15 reps The contralateral leg was not trained Cuffs were applied to the thigh	**Muscle strength:** **-**5 RM and estimated 1 RM of leg press **-**Max isometric knee extensors and flexors strength **Function:** TUG 30 STS 40m fast-paced walk test knee ROM	Muscle strength: **1 RM leg press:** BFR significantly better in **pre-op 3m-post** but not in 12m-post With large effect sizes at all time points **1 RM knee extension:** BFR significantly better in **all time points** than the control group, with a large effect size in all time points **Max iso knee ext:** BFR only sig better at **3m-post op,** moderate to large **effect size in all time points,** favoring BFR **Max iso knee flex:** **No difference between groups,** but **a moderate to large effect size** favoring BFR at all time points Function: -None between group difference at any time point -Effect size **30 STS** favoring BFR **pre-op**, moderate effect size **TUG** at **3m-post op** -At **12 m Post**, large Effect sizes favoring **CON** emerged for **30 STS**, **TUG,** and **40mFWT** **-Adverse event: None**
[32]	Non-blinded Randomized controlled trial	**Location of the study:** USA **Enrollment period:** NR	***n* = 10** (total), ***n* = 4** (control) and ***n* = 6** (BFR group) **mean age**: 67.2 ± 7.1 years **sex distribution:** 7 females and three males **Inclusion criteria:** -age: 60 to 75 years -scheduled for unilateral TKA for knee OA	**4 weeks before surgery** **The control group**: no exercise **The BFR group**: 2 to 3 sessions a week with a minimum of 8 sessions and a maximum of 12 (one patient did only six sessions) **Assessment time points:** Baseline: 4 to 5 weeks pre-op 2w-post op	2 to 3 sessions a week -Brief warm-up **THEN** Leg press, leg extension, leg curl, and calf extension at an intensity of **30% of 1 RM** Exercises were performed to volitional fatigue (**no standardized reps**) -Cuff pressures for each An automated cuff inflator determined the individual depending on systolic blood pressure and thigh circumference -Cuffs remained inflated during exercise, but they were deflated for a 3 min rest between exercises. -Cuffs were applied to the thigh	**Muscle strength:** Quadriceps Peak torque strength **Function:** SPPB 6 MWT	**Muscle strength:** **Peak torque**: Similar decrease in both groups **Function:** **SPPB**: after 2 weeks- post op, both groups decreased values, but a greater decrease in the control group **6 MWT**: similar decrease **Adverse event: NR**
[33]	Single-blinded randomized controlled trial	**Location of study:** Japan **Enrollment period:** from September 2019 to December 2021	***n* = 22** (total), ***n* = 11** (BFR group) and ***n* = 11** (LST group) **mean age:** 73 years **sex distribution:** 4 males and 18 females **Inclusion criteria:** Age between 60 and 79 years, with an advanced knee OA condition scheduled for unilateral TKA	**4weeks before surgery** **Both groups** had a 10 min warm-up, and each session lasted 50 min, including resistance and aerobic exercises **The LST group:** 6 ± 3 training sessions before surgery, and performed the **same exercises** as the BFR group while using two cuffs inflated at only 20 mmHg on the thigh and on the calf **The BFR group**: 7 ± 2 training sessions before surgery **Surgery:** tricompartmental uncemented TKA using a low-contact-stress implant with Tourniquet use at 300 mmHg **After surgery**, both groups received the same inpatient and outpatient rehabilitation treatment **Assessment time points:** Baseline: 6w preop Multiple preop and postop assessments until 3 months	2 to 3 sessions a week -Squats, forward lunges using body weight for resistance, and seated bilateral knee extensions at **30% of the maximum isometric voluntary contraction.** The exercise consisted of three sets of 10 reps, with each rep including 3 s each of eccentric, isometric, and concentric phases There was a 30 s rest between sets and a 60 s rest between different exercises -Cuffs were inflated at 100–120 mmHg on the thigh and calf	**Muscle strength:** isometric quadriceps strength **Function:** 30 STS TUG SCT	**Muscle strength:** **Quadriceps strength:** no significant differences in the rate of increase in the quadriceps strength before and after the intervention, or in the rate of reduction in quadriceps strength before and after surgery between the groups **Function:** no difference between the two groups concerning the 30 STS, TUG, and SCT after the intervention, nor after the surgery **Adverse event: None**

## Data Availability

The data that support the findings of this study are publicly available. Further data are available from the corresponding author upon reasonable request.

## Supplementary Materials

The following supporting information can be downloaded at: https://www.mdpi.com/article/10.3390/medicina61101879/s1, Table S1: List of excluded full-text records with reasons for exclusion.
