# Supplementary material for: Blood Flow Restriction Training in Knee Arthroplasty: A Systematic Review of Current Evidence on Postoperative Muscle Strength and Function
Source: Medicina (Kaunas). 2025 Oct 20;61(10):1879. doi: 10.3390/medicina61101879 (PMC12566041; doi:10.3390/medicina61101879)
Supplement: Supplementary file 1 [file medicina-61-01879-s001.zip › medicina-3866177-supplementary.pdf]

Supplementary Table S1: List of excluded full-text records with reasons for exclusion

No.	Author / Year	Title / Source	Reason for Exclusion
1	ClinicalTrials.gov (2020)	BFRT vs Standard PT After Total Knee Arthroplasty (NCT04366336)	Ongoing trial, no published results available
2	Hughes et al. (2018)	Blood flow restriction training as a prehabilitation concept in total knee arthroplasty: A narrative review about current preoperative interventions and the potential impact of BFR	Narrative review, not an interventional RCT
3	Kacin et al. (2021)	Blood Flow Restriction Training Can Improve Peak Torque Strength in Chronic Atrophic Postoperative Quadriceps and Hamstrings Muscles	Postoperative population, not prehabilitation before surgery
4	Stevens-Lapsley et al. (2024)	Can blood flow restriction therapy improve quality of life and function in dissatisfied knee arthroplasty patients?	Focus on dissatisfied/revision patients rather than primary TKA prehabilitation
5	Naylor et al. (2022)	Does Blood Flow Restriction Therapy Improve Leg Strength in Patients With a Painful Total Knee Arthroplasty?	Symptomatic postoperative patients, not preoperative intervention
6	Scott et al. (2023)	Editorial: Clinical application and impact of blood-flow-restriction training	Editorial, lacks original data
7	Li et al. (2025)	Preoperative blood flow restriction training combined with postoperative continuous passive motion in elderly patients after total knee arthroplasty: study protocol for a randomized controlled trial	Study protocol only; no results reported
8	Jakobsen et al. (2020)	Efficacy of low-load blood flow restricted resistance EXercise in patients with Knee osteoarthritis scheduled for total knee replacement (EXKnee): Protocol for a multicentre RCT	Protocol, no outcomes yet published
9	Garcia et al. (2024)	Exercise with blood flow restriction among adults undergoing total knee arthroplasty: A scoping review	Scoping review, not RCT
10	Ladlow et al. (2019)	Home-Based Exercise With Blood Flow Restriction to Improve Quadriceps Muscle and Physical Function After Total Knee Arthroplasty: A Case Report	Case report, single patient
11	Skoffer et al. (2024)	Is preoperative blood flow restriction exercise superior to preoperative usual care treatment before total knee arthroplasty …	Combined perioperative intervention; outside strict prehabilitation window

12	Hughes et al. (2023)	Knee Loading With Blood Flow Restriction Can Enhance Recovery After Total Knee Arthroplasty	Postoperative loading intervention, not preoperative
13	Skoffer et al. (2024)	Preoperative low-load blood flow restricted exercise induces persistent gains in knee extensor strength …	Secondary analysis, duplicate sample population
14	Holm et al. (2024)	Quadriceps Strength Loss Following Total Knee Arthroplasty as a Predictor of Three-Month Strength Recovery	Observational / secondary analysis, not intervention
15	Garcia et al. (2025)	Exercise with blood flow restriction among adults undergoing total knee arthroplasty	Duplicate of review / non-RCT
16	Cochrane Library (2023)	The effects of Blood Flow Restriction training after total knee arthroplasty	Registry record or database entry, no published results
17	Johnson et al. (2024)	Utilization of Blood Flow Restriction Therapy with a Former Triathlete After Total Knee Arthroplasty: A Case Report	Case report of one subject
18	Clarkson et al. (2020)	Blood flow restriction exercise post knee replacement: A systematic review of safety and efficacy	Systematic review, not a primary RCT
